# Cataplexy Mistaken for Seizures in a Patient With Undiagnosed Narcolepsy Type I

**DOI:** 10.7759/cureus.57540

**Published:** 2024-04-03

**Authors:** Erafat D Rehim, Martina Vendrame

**Affiliations:** 1 Neurology, Emory University, Atlanta, USA; 2 Sleep Medicine/Epilepsy, Lehigh Valley Fleming Neuroscience Institute, Allentown, USA

**Keywords:** atonia, narcolepsy misdiagnosis, cataplexy, epileptic seizures, narcolepsy type 1

## Abstract

Narcolepsy Type 1 is a sleep disorder, with cataplexy as its cardinal feature, characterized by sudden decrease or loss of muscle tone triggered by strong emotions. Cataplexy can be misdiagnosed as epileptic seizures given its clinical similarity to atonic seizures. The low prevalence of the disease added another layer of complexity in providing timely and accurate diagnosis. We report a case of a young man with recurrent episodes of falling and an inability to respond, initially misinterpreted as epileptic seizures due to findings in routine electroencephalography (EEG). Anti-seizure medications were ineffective, and subsequent ambulatory EEG revealed no epileptic activity during events. A detailed history uncovered symptoms of cataplexy and daytime sleepiness, leading to the correct diagnosis of narcolepsy type I confirmed by polysomnogram (PSG) and mean sleep latency test (MSLT). Discontinuation of anti-seizure medications and treatment with venlafaxine successfully resolved cataplexy. The case highlights the importance of a thorough clinical history in distinguishing cataplexy from seizures, as well as the caution against relying solely on EEG findings for epilepsy diagnosis. Ambulatory EEG can help exclude epileptic events, and PSG with MSLT are necessary to confirm narcolepsy type I.

## Introduction

Narcolepsy Type 1 (Na-1) is a primary sleep disorder, manifested as excessive daytime sleepiness, sleep paralysis, hypnagogic hallucinations, and cataplexy [1]. Na-1 is uncommon, with an estimated prevalence of 0.02-0.05% in the United States [2]. Cataplexy is a cardinal symptom of Na-1, characterized by rapidly progressive loss of skeletal muscle tone, often triggered by positive emotions [1]. The severity of cataplexy can vary from mild muscle weakness to a complete loss of muscle control, resulting in falling to the ground. Although the person remains fully conscious and aware during the episode, they are unable to move and they may be unable to speak. It remains a challenging task to provide an accurate and timely diagnosis, which usually requires detailed history taking, evaluation of sleep diary, and data from polysomnography (PSG), actigraphy, multiple sleep latency testing (MSLT), among others. The stereotypical nature of the spells resembles atonic seizures and syncopes, in combination with their low prevalence, put patients at risk for misdiagnosis [3-5], which leads to delays in diagnosis and unnecessary investigations or treatments. Here, we present a case of a 22-year-old man with cataplectic attacks which were misdiagnosed as epileptic seizures. A comprehensive history led to the suspicion of cataplexy. Appropriate testing confirmed the correct diagnosis.

## Case presentation

A 22-year-old man was referred to our clinic after being hospitalized for “new onset seizures”. Episodes of presumed epileptic etiology started about four months prior, they were described as “syncopal events” and occurred one to two times per week. He had normal birth and development and no family members with a history of similar symptoms. He had no history of seizures, myoclonic jerks, or other neurological complaints. He reported previously taking venlafaxine for around two years to address mild symptoms of depression and anxiety but discontinued it six months ago. His body mass index (BMI) was 27 Kg/m^2^. His neurological exam was normal.

During his hospital stay, his brain MRI showed normal findings and a routine electroencephalogram (EEG) showed one isolated generalized ~4 Hz spike and wave during drowsiness. He was diagnosed with epilepsy and was started on levetiracetam 500 mg twice a day. About one month later, he reported no change in the frequency of the events. Levetiracetam was increased to 750 mg and a three-day-ambulatory EEG was obtained. EEG recording captured one typical event that did not have any electrographic correlation, except for movement and myogenic artifact. There were no associated EKG changes. The recording did not capture any epileptiform abnormality.

**Figure 1:**
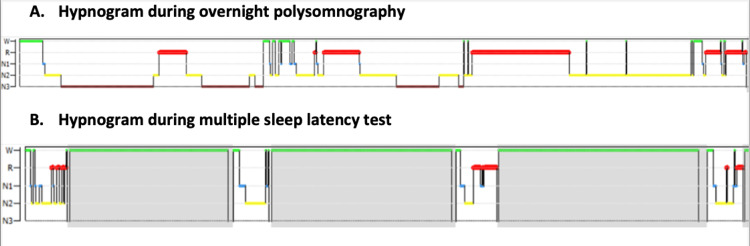
Results of sleep studies-hypnogram Hypnogram during (A) overnight polysomnography and (B) multiple sleep latency test. Normal sleep architecture and rapid eye movement (REM) sleep periods were seen overnight (A). Sleep was captured during four naps opportunities, with REM seen during three of the four naps (B). W: wakefulness; R: REM sleep (red color); N1: stage 1 sleep; N2: stage 2 sleep; N3: stage 3 sleep. Image credit: Martina Vendrame.

On further questioning, the patient reported that those events primarily occurred while walking in his yard or during college classes and that his last episode had occurred while watching his father’s favorite show “Stephen Colbert’s Late-Night Show”, he had no recollection of events occurring in the absence of an emotional trigger. He described that he was aware of collapsing to the floor, most times after a sensation of extreme tiredness in his legs. He was able to understand what bystanders were telling him, but he could not respond to questions and was unable to follow simple directions. There were no associated cardiovascular or respiratory symptoms. Episodes lasted for a few minutes, after which he was able to stand back up and felt “fully awake”. Taking together the description of the events and the ambulatory EEG findings, we concluded that his episodes were highly suggestive of cataplexy. Suspecting narcolepsy, we inquired about sleepiness and other narcolepsy-related symptoms. He reported that he had always been “sleepy” but he himself always related to his high-performance requirements and his tendency to be a “night owl”. He never had nocturnal hallucinations but reported episodes of likely sleep paralysis during his high school years. He disclosed that he loved taking “power naps” during the daytime and found online “on-demand” college classes ideal for incorporating these naps into his academic day. His sleep diary showed regular nocturnal sleep periods between 12 midnight to 6 am and at least one 1-hour nap per day. Epworth Sleepiness Scale (ESS) was 16. His polysomnogram (PSG) showed normal findings. On the mean sleep latency test (MSLT), the mean sleep latency was 2.9 minutes across four naps with three sleep-onset rapid eye movement (REM) periods (SOREMPs), confirming the diagnosis of Na-1 (Figure 1). Levetiracetam was stopped, and he was restarted on venlafaxine extended release 75 mg daily, together with modafinil 100 mg daily. After four weeks, venlafaxine was increased to 150 mg daily providing resolution of cataplexy attacks. At his three-month follow-up, he reported no further events of cataplexy and feeling less sleepy but taking occasional “power naps”.

**Table 1:** Result of multiple sleep latency test (MSLT) *Mean sleep latency in healthy adults: 10–15 minutes; ^$^Mean rapid eye movement (REM) onset in healthy adults: 80-100 minutes. REM onset within 15 minutes of sleep onset is defined as sleep onset rapid eye movement periods (SOREMPs) [6].

	Nap 1	Nap 2	Nap 3	Nap 4
Sleep latency (minutes)*	2.7	3.4	2.1	3.3
REM onset latency (minutes)^$^	11.0	-	7.0	7.0

## Discussion

**Figure 2:**
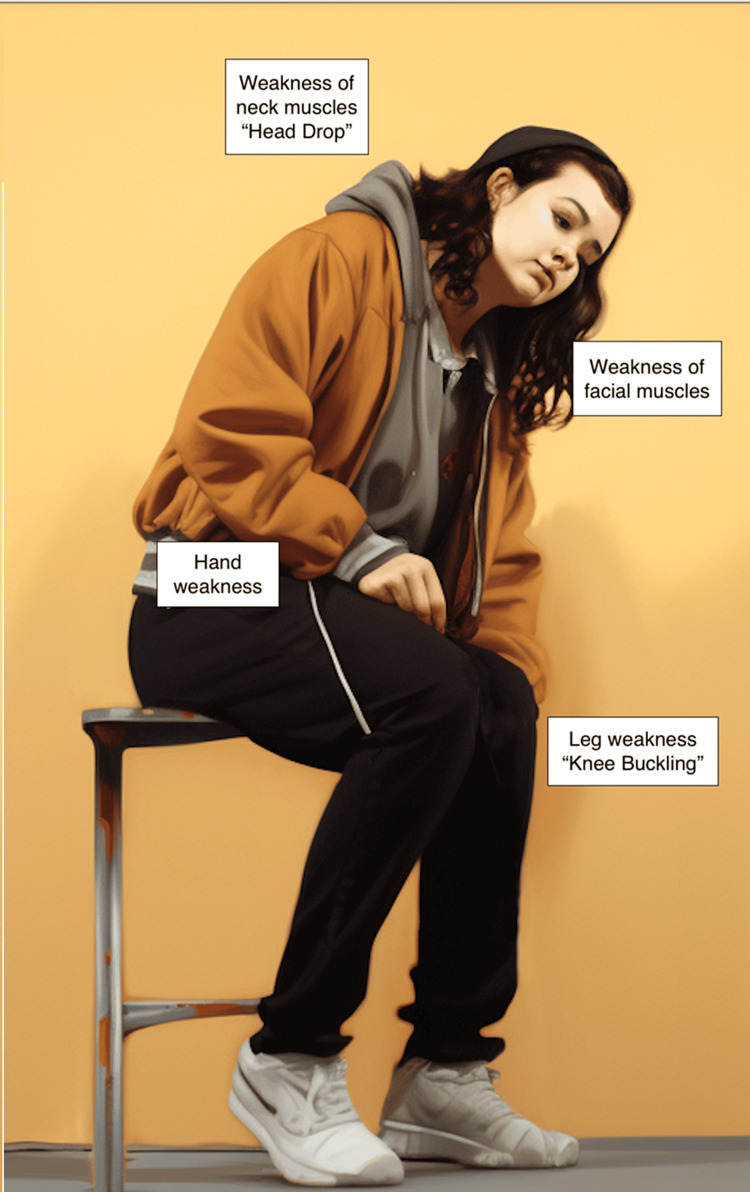
Clinical features of partial cataplexy Patients with partial cataplexy may present with subtle signs resulting from partial atonia of different muscle groups. Loss of muscle tone of neck muscles may result in head drop, while weakness of face muscle may cause dysarthria. Hand weakness may result in temporary loss of dexterity and leg weakness may force the patient to sit down and rest. This image was generated by Erafat Rehim and Martina Vendrame using the artificial intelligence (AI) art generator Midjourney (www.midjourney.com).

Cataplexy is a sudden and temporary muscle weakness or paralysis triggered by strong emotions, typically laughter, joy, excitement, or anger. During a cataplectic episode, an individual's voluntary muscles suddenly lose their tone, leading to weakness or even complete collapse. The severity of cataplexy can vary from mild muscle weakness to a complete loss of muscle control, resulting in falling to the ground [1]. Cataplexy can be partial, affecting only face muscles, hands, truck and/or limbs (Figure 2). With partial weakness of face muscles, cataplexy may present with the complaint of inability to speak out in response to questions. When weakness affects muscles of the trunk and limbs, the individual may be unable to move on command. Others have reported difficulty in distinguishing between cataplexy and seizures [4,7]. Cataplexy never affects respiratory muscles. The person remains fully conscious and aware during the episode and is able to recall the whole episode in detail. Table 2 presents the main clinical features differentiating cataplexy from epileptic seizures. 

**Table 2:** Clinical features differentiating cataplexy from epileptic seizures Table Credit: Martina Vendrame

Clinical feature	Cataplexy	Epileptic seizure	Questions
Lack of awareness	Not present	Present or not present	Did you pass out? What do you recall about the event? Did you recall people asking you questions?
Loss of muscle tone (partial or complete)	Present	Present or not present	Did the patient fall abruptly or lose limbs muscle strength?
Speech arrest	Present or not present	Present or not present	Could you talk, or answer questions?
Respiratory distress	Not present	Present or not present	Did you have trouble breathing?

Cataplexy is specifically associated with Na-1 [6]. People with narcolepsy may experience excessive daytime sleepiness, sleep paralysis and hallucinations during the transition between wakefulness and sleep. It is, therefore, imperative to investigate whether these symptoms have been present but overlooked. Narcolepsy develops most commonly during adolescence, during which daytime sleepiness may be attributed to the tendency to delay the sleep phase and self-induced sleep deprivation. Other conditions associated with cataplexy are neurodevelopmental conditions such as Niemann-Pick Type C disease and Coffin-Lowry syndrome, which are typically diagnosed in young adulthood [1].

Cataplexy is thought to be the result of intrusion of incomplete REM into wakefulness [8]. Activation of the same brain stem regions occurs in both REM sleep atonia and cataplexy. The sublaterodorsal nucleus (SLD), a group of neurons in the caudal pons, plays a significant role in REM sleep. Glutamatergic neurons in the SLD project to the ventromedial medulla, stimulating GABAergic and glycinergic interneurons, resulting in the atonia seen during REM sleep. In cataplexy, projections from the amygdala to the SLD reduce muscle tone when processing positive emotional stimuli, resulting in muscle atonia and postural failure. 

Treatment of cataplexy has primarily revolved around the administration of antidepressant medications [6]. The initial antidepressants demonstrated to be effective were tricyclic antidepressants. Among the tricyclic antidepressants frequently prescribed for cataplexy are imipramine, protriptyline, and clomipramine. While selective serotonin reuptake inhibitors (SSRIs) have also found application, they necessitate relatively elevated dosages, leading to heightened undesirable reactions. Optimal outcomes with minimal adverse effects might be achievable through the utilization of low to moderate dosages of serotonin-norepinephrine reuptake inhibitor (SNRI) antidepressants [9]. Thus, when cataplexy is suspected, it is essential to review the patient's medical history to identify any medications that could have exacerbated cataplexy symptoms when discontinued.

Another factor that contributed to the misdiagnosis of epilepsy was the presence of one single epileptiform abnormality in the patient's EEG. Epileptiform abnormalities can be found in EEG recordings of healthy individuals with no seizures or epilepsy. Studies on military recruits and other healthy pediatric and adult populations have reported epileptiform abnormalities in 1.3 to 3% of the subjects [10]. Thus, this case reminds us of the necessity for clinical correlation whenever epileptiform abnormalities are identified on EEG. Although certain EEG patterns may suggest specific epilepsies and/or epilepsy syndromes, the presence of seizures always needs to be carefully investigated. Ultimately, it is important to recognize that our primary goal is treating the patients, rather than exclusively concentrating on the results of EEG tests. This emphasizes the necessity of integrating clinical contexts with EEG findings when formulating diagnostic hypotheses. In our case, we cannot exclude that this patient will develop epilepsy later in life. 

EEG monitoring capturing a typical event confirmed the incorrect diagnosis of seizures. EEG monitoring is widely used in clinical practice to rule out non-epileptic events, and in case of uncertain diagnosis of cataplexy, it may be of use to rule out epileptic events. Cataplexy remains a clinical diagnosis, but when narcolepsy is suspected, testing with MSLT is crucial in determining the diagnosis. The MSLT involves a series of daytime nap opportunities that are administered in a controlled sleep laboratory environment following an overnight PSG, which helps exclude other sleep disorders. During the MSLT, patients are monitored for their ability to fall asleep quickly and enter REM sleep, which is a hallmark feature of narcolepsy. The intervals between these naps, known as "latency periods," are measured to gauge the time taken to fall asleep. A shorter latency period, particularly the tendency to rapidly enter REM sleep, is indicative of narcolepsy.

## Conclusions

This article reports the case of a young man with recurrent episodes of falling and an inability to respond, initially misinterpreted as epileptic seizures due to findings in routine EEG. Cataplexy may mimic seizures. It is critical not to rely solely on isolated epileptiform discharges on EEG to diagnose epilepsy. A thorough history could help differentiate the two entities, while PSG with MSLT remains essential to confirm the diagnosis of narcolepsy.
